# Clinical and Dermoscopic Features of Melanocytic Lesions on the Face Versus the External Ear

**DOI:** 10.5826/dpc.1104a124

**Published:** 2021-10-01

**Authors:** Teresa Deinlein, Andreas Blum, Günter Schulter, Holger A. Haenssle, Ralph Braun, Roberta Giuffrida, Rainer Hofmann-Wellenhof

**Affiliations:** 1 Department of Dermatology, Medical University of Graz, Graz, Austria; 2 Public, Private, and Teaching Practice, Konstanz, Germany; 3 Department of Psychology, Biological Psychology Unit, Karl-Franzens-University Graz, Graz, Austria; 4 Department of Dermatology, University of Heidelberg, Heidelberg, Germany; 5 Department of Dermatology, University of Zürich, Zürich, Switzerland; 6 Department of Clinical and Experimental Medicine, Dermatology, University of Messina, Messina, Italy

**Keywords:** nevi, melanoma, dermoscopy, face, ear

## Abstract

**Introduction:**

Melanoma of the external ear is a rare condition accounting for 7–20% of all melanomas of the head and neck region. They present classical features of extra-facial melanomas clinically and dermoscopically. In contrast, facial melanomas show peculiar patterns in dermoscopy.

**Objectives:**

To evaluate whether there are clinical and/or dermoscopic differences in melanocytic lesions located either at the external ear or on the face.

**Methods:**

In this retrospective study we reviewed an image database for clinical and dermoscopic images of melanomas and nevi located either on the face or at the level of the external ear.

**Results:**

65 patients (37 men; 63.8%) with 65 lesions were included. We found no significant differences in comparing face melanomas with melanomas at the level of the external ear, neither clinically nor dermoscopically. However, we provided evidence for differences in some clinical and dermoscopic features of melanomas and nevi of the external ear.

**Conclusions:**

In this study, we reported no significant differences in comparing melanomas on the face with melanomas of the external ear, both clinically and dermoscopically. Furthermore, we provided data on clinical and dermoscopic differences comparing nevi and melanoma of the external ear.

## Introduction

**Figure 1:**
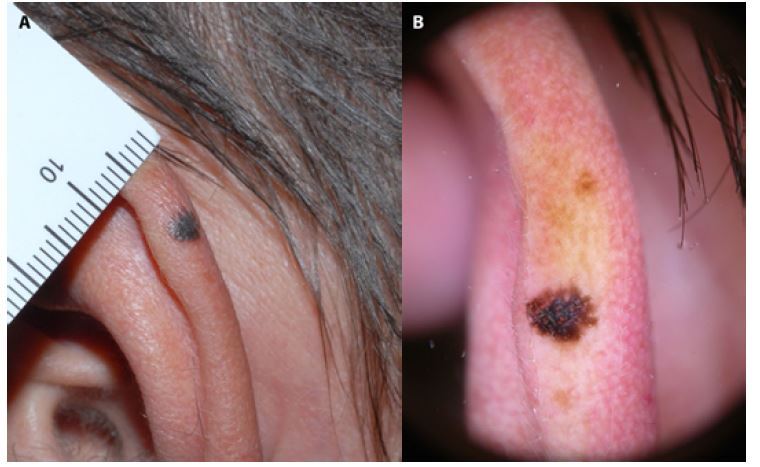
(A) Clinical and (B) dermoscopic image of a melanoma of the external ear. One can observe a dark brownish to black-coloured, well demarcated lesion clinically. It exhibits an irregular pigment network and shiny-white streaks in dermoscopy. Histopathology reported a malignant melanoma with a tumor thickness of 0.4 mm.

Melanoma of the external ear (MEE) is a rare condition, and its prognosis is still a matter of debate as long-term data are lacking. The external ear is considered as a special location as the skin is thin and lymphatic drainage is unpredictable. About 25% of all cutaneous melanomas are located at the head and neck region and among those, 7–20% are located on the external ear [1–4]. Querying the Surveillance, Epidemiology, and End Results registry (SEER-registry) reveals 76,380 new diagnosed cases of MEE in 2016 in the United States. 2 population-based analyses suggest surgical removal as the treatment of choice; however, following recent data, wider surgical margins for melanomas of the external ear do not affect the overall survival [3,4]. Reviewing current literature shows, that melanomas of the external ear exhibit classical features of extra-facial melanomas, both clinically and dermoscopically (eg atypical pigment network, irregular dots and/ or globules, and peripheral streaks) (Figure 1, A and B) [5].

**Figure 2:**
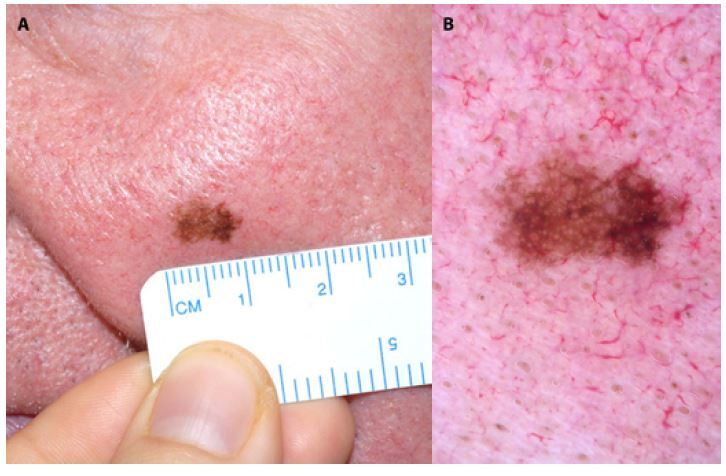
(A) Clinical (A) and (B) dermoscopic features of a melanoma on the left cheek. Clinically, a light-to dark brown, poorly demarcated macule is evident. The lesion shows different shades of brown, asymmetric pigmented follicular openings, and some concentric circles. Histopathological report revealed an in-situ melanoma.

In contrast, melanomas of the face are known to be associated with chronic sun-damaged skin and considered as slow-growing tumours. Their incidence increases mostly due to the higher cumulative exposure to ultraviolet radiation [5–7]. Stolz et al [6], first described a progression model from in-situ melanomas of the face (lentigo maligna) to dermoscopically invasive ones. At initial stages asymmetric pigmented follicles and grey dots around the follicles are observable. As the tumour progresses rhomboidal structures and finally homogenous pigmented areas and obliteration of the follicles are seen (Figure 2, A and B). Sensitivity and specificity of these features are reported to be 89% and 93% respectively [5–7].

The aim of this study was to evaluate whether there are clinical and/or dermoscopic differences in melanocytic lesions located either at the external ear or on the face.

## Methods

This was a retrospective observational single-centre study conducted at the Department of Dermatology in Graz to evaluate whether there are clinical and/or dermoscopic differences in melanocytic lesions located either at the ear or in the face. The study was conducted over a 1-year period. Ethics’ committee approval was waived since we did not affect the standard care of patients and data were anonymized. Research was performed following the principles of the declaration of Helsinki. We retrospectively reviewed the image database from the Department of Dermatology in Graz for all clinical and dermoscopic images of melanomas (histopathologically proven) and nevi (histopathologically proven or unchanged in a follow-up over 6 months) located either on the face or at the level of the external ear. For 7 cases, there were no clinical images as follow-ups with sequential dermoscopy alone were performed.

Clinical images were obtained using a 10.2-megapixel digital camera (D200, Nikon Corporation Tokyo, Japan) and dermoscopic images were acquired with a 4-megapixel digital camera (Nion Coolpix 4500, Nikon, New York, USA) equipped with a contact polarized dermoscope (DermLite Photo 3Gen, California, USA). Due to comparison reasons, we matched each group of melanomas to contain almost the same number of patients. Patients’ demographics (sex and age at diagnosis) and tumor information (anatomic site and histological diagnosis) were summarized in an Excel file.

All clinical and dermoscopic images were subsequently uploaded in the web-based database of the International Skin Imaging Collaboration (ISIC archive). This upload was necessary as 5 experts evaluated the images from different departments over Europe. Experts had full access to the images and were blinded for tumor data.

Lesions were reviewed and analyzed following the clinical and dermoscopic criteria based on the third consensus conference of the International Dermoscopy Society from 2016 [11]:

- Colour clinically: Black, brown, grey-blue, light-brown, red, and white.
- Face-specific dermoscopic features: Asymmetric pigmented follicular openings, pseudonetwork, annular-granular pattern/grey dots, circle within a circle/concentric circles.
- Melanoma criteria in general: Irregular globules, scar-like depigmentation, tan peripheral structureless area, atypical pigment network, blue-white veil, milky-red areas, pseudopods, regular globules.

Statistical analyses were performed calculating 2 unifactorial analyses of variance (one-way ANOVA) with the clinical and dermoscopic criteria as independent variables and the mean ratings of the 5 experts as the dependent variables using the latest SPSS software (version 23; IBM, Armonk, NY, U.S.A). Expert ratings varied from 0 (none of the five experts diagnosed a specific feature) to 5 (all 5 experts observed a single feature) in a specific patient. Comparison of means of these expert ratings across all patients was then done by analyses of variance.

Moreover, we also estimated the reliability of clinical and dermoscopic judgements, ie, the inter-rater repeatability using intraclass correlations (SPSS: Two-way mixed model testing absolute agreement of single raters). These correlation measures are reported in Table 1 and reflect the variation between the raters evaluating the same criterion in the same group of 65 patients examined in our study. The higher the coefficients, the higher was the consistency of ratings concerning a certain clinical or dermoscopic feature. Values lower than 0.5 are indicative of a poor reliability, ie, experts substantially differ in their ratings of that feature.

## Results

### Demographics and Lesions’ Characteristics

A total of 65 patients (37 men; 63.8%) with 65 lesions were included. The mean age at presentation was 48 years (range 8–92 years). 43 lesions (66.2%) were located at the ear and 22 on the face (33.8%). The localisation of the lesions in the face scattered as follows: 12 lesions were located on the cheek, 4 lesions on the nose, 3 pre-auricular lesions, 2 lesions at the level of the temple, and 1 lesion on the chin. The distribution of the lesions affecting the different parts of the ear showed the following: 26 lesions were located at the helix, 6 lesions on the earlobe and the back of the auricle respectively, 4 lesions on the antihelix, and 1 lesion on the antitragus.

47 lesions (72.3%) were histopathologically diagnosed as melanomas (8 in-situ melanomas and 39 invasive melanomas) with a mean tumour thickness of 1.21mm (range 0.13mm–6.0mm). Among those, 25 lesions (53.2%) were located on the ear, showing a mean tumour thickness of 1.47mm and 22 (46.8%), on the face with a mean tumour thickness of 0.87mm. 18 lesions were - due to not observable changes during follow-up - diagnosed as nevi; all of these were located at the level of the ear.

### Co-Occurrences of Clinical Features Irrespectively of Tumors’ Localization

A total of 58 clinical images was evaluated based on the abovementioned criteria and revealed the following significant co-occurrences of clinical colours:

- Black colour and grey-blue colour (r=0.54, p < 0,001);
- Brown colour and light-brown colour (r=0.64, p <0,001);
- Grey-blue colour with white and red colour (r=0.34, p <0,01 and r=0.43, p < 0,001 respectively);
- Red colour with white colour (r=0.41, p <0,001).

#### Comparison of Clinical Features in Melanomas on the Ear vs. Nevi on the Ear

A significant difference concerning clinical features when comparing these groups could be established. Diagnoses of the clinical feature “colour gray-blue” were more than twice frequent in melanomas than in nevi (mean expert ratings: 0.45 vs. 0.21; F_(1/34)_=4.11; p=0.05).

#### Comparison of Clinical Features of Melanomas on the Face vs. Melanomas of the Ear

No significant differences could be recognized concerning clinical features in these groups.

### Co-Occurrences of Dermoscopic Patterns Irrespectively of Tumors’ Location

A total of 65 dermoscopic images was evaluated concerning face-specific and general criteria of melanomas. The following significant co-occurences of patterns were observed:

- Asymmetric pigmented follicles with scar-like depigmentation, annular-granular structures, tan peripheral structureless areas and concentric circles (r=0.52, p <0.001; r=0.56, p <0.001; r=0.36, p <0.003 and r=0.38, p <0.004 respectively).
- Scar-like depigmentation with annular-granular structures, tan peripheral structureless areas, concentric circles, blue-white veil and milky-red areas (r=0.34, p <0.006; r=0.45, p <0,001; r=0.32, p <0.009; r=0.27, p <0.027 and r=0.27, p <0.032 respectively).
- Annular-granular structures with concentric-circles (r=0.3, p <0.016) and negative correlation of annular-granular patterns with regular globules (r=−0.015, p <0.015)
- Tan peripheral structureless areas with concentric circles and atypical pigment network (r=0.32, p <0.008 and r=0.25, p <0.04).
- Atypical pigment network with a blue-white veil (r=0.32, p <0.009).
- Blue-white veil with shiny-white structures and milky-red areas (r=0.28, p <0.022 and r=0.42, p <0.001).
- Negative correlation of regular globules with asymmetric pigmented follicles and tan peripheral structures (r=−0.28, p <0.021 and r=−0.27, p <0.027).

#### Comparison of Dermoscopic Features of Ear Melanomas Versus Nevi on the Ear

We found some dermoscopic patterns to be significantly different between melanomas and nevi at the ear: asymmetric pigmented follicles, scar-like depigmentation, annular-granular patterns, tan peripheral structureless areas and regular globules; detailed results are provided below (Table 2).

#### Comparison of Dermoscopic Features of Face Melanomas Versus Ear Melanomas

No significant differences could be proven concerning dermoscopic features in comparing these groups.

## Discussion

In this retrospective study including 65 patients we did not find any significant clinical or dermoscopic differences when comparing melanomas on the face and melanomas of the external ear. To the best of our knowledge, this is the first study providing data on this topic. These results somewhat contradict previous knowledge as skin’ s structure and texture at the external ear and on the face seem to be very similar. Consequently, face-specific criteria for melanomas should be valid and be used also for melanomas of the external ear. Of note, the limitation of this study relies on the fact that most of the included melanomas were invasive while just 8 tumors were in-situ melanomas. It is well known that – as melanomas progress – one can find similar clinical and dermoscopic features irrespectively of the tumor’s localization [5,9,11–13].

**Figure 3 A–D:**
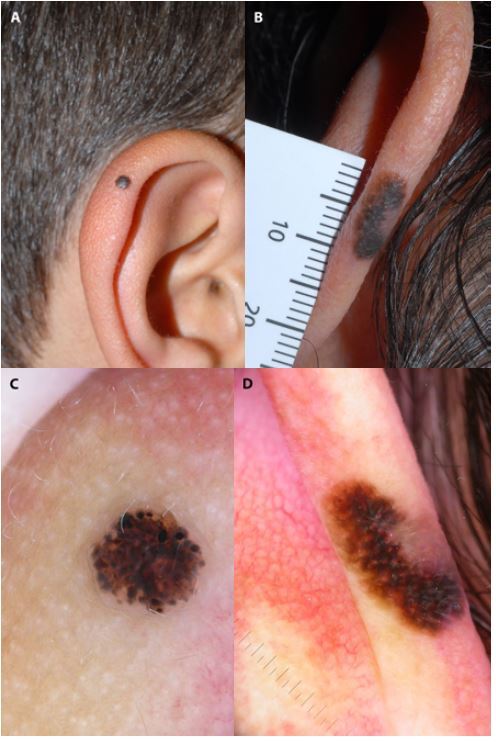
Two clinical images comparing a nevus (A) and a melanoma (B) of the external ear. The nevus presents as roundish, well-demarcated, dark-brown plaque, whereas the melanoma presents as oval-shaped lesion showing different shades of brown, black and whitish-blue areas. Histopathology reported a tumour thickness of 0.6mm. Respective dermoscopic images are shown in the lower line. The nevus (C) exhibits regular distributed brownish and black globules. The melanoma (D) shows different shades of brown colour and black as well as whitish-blue areas.

Moreover, we found significant differences in clinical and dermoscopic patterns when comparing melanomas and nevi on the external ear. Melanomas of the external ear exhibited the clinical feature “colour gray-blue” more than twice frequent when compared to nevi on the ear (Figure 3, A–D). This is in line with data from the literature, as gray-blue colour is considered as a strong clue for melanoma [5,9,11,12]. Moreover, we presented a number of dermoscopic features differing between melanomas and nevi of the ear respectively (Table 2). These results are again in line with current data as asymmetric pigmented follicles, scar-like depigmentation, annular-granular structures and tan peripheral structureless areas were significantly more frequent observed in melanomas, whereas regular globules typically indicate nevi [9,11,12]. The early differentiation of melanocytic lesions at the external ear is of clinical relevance. A large study [14] comparing melanomas of the external ear and the head-/neck-region in over 130,000 patients proved that ear melanomas were an independent factor for tumor stage I and invasive behaviour. Furthermore, the authors found that men had a higher likelihood to develop MEE when compared to women.

Furthermore, significant co-occurrences among different dermoscopic patterns regardless of tumors’ localization were observed. Notably, all positive correlations were found between face-specific and extra-facial criteria for melanoma. In addition, we found a number of negative correlations in this group. Regular globules were negative correlated with asymmetric pigmented follicles and tan peripheral structures (p <0,021 and p <0,027, respectively); furthermore, a negative correlation between annular-granular patterns and regular globules could be proven (p <0,015). These results are well-established knowledge as regular globules in dermoscopy indicate a benign nevus whereas asymmetric pigmented follicles, tan peripheral structures, and the annular-granular pattern are strong indicators for melanomas [9,11–13]. From this one can infer that the presence of dermoscopic patterns considered as “benign” excludes a melanoma and the other way round.

Our study has several limitations. First, due to the retrospective setting of our study, the interpretation of results is limited. Second, we did not include nevi of the face and can therefore not provide data about this entity in comparison to investigated entities. However, there is evidence that nevi of the face are mainly congenital and, especially in older individuals (>51 years), mostly present as raised, palpable, and hypopigmented lesions [13]. Third, the mean tumor thickness of melanomas on the face versus the external ear differed substantially (0,87 mm versus 1,47 mm). Therefore, comparison of these melanoma-types is limited. Fourth, we did not include information about the total number of nevi, personal or family history of melanoma or degree of photo damage.

To conclude, our study for the first time described that there are no significant differences when comparing melanomas of the face and melanomas of the external ear, clinically and dermoscopically. These results indicate that face-specific criteria for melanomas are also valid for melanomas of the external ear and vice versa. Furthermore, our study for the first time provided data on clinical and dermoscopic differences in comparing nevi and melanoma of the external ear.

**Key messages**

- Melanomas of the external ear and face melanomas differ in terms of their prevalence, clinical, dermoscopic appearance, and biological behaviour. Prognosis of ear melanomas is still a matter of debate. It is common knowledge that ear melanomas usually exhibit the classical features of extra-facial melanoma.
- To date, no study formally investigated possible clinical and/or dermoscopic differences in melanocytic lesions on the face versus the external ear. The results of this study show no significant differences when comparing melanocytic lesions on the face versus the external ear, thus indicating that face-specific criteria for melanomas are also valid for melanomas of the external ear and vice versa. Furthermore, data on clinical and dermoscopic differences comparing nevi and melanoma of the external ear are here reported for the first time.

## Figures and Tables

**Table 1:** Intra-class correlations of expert ratings of clinical and dermoscopic features. The left column shows the evaluated clinical (c) and dermoscopic (d) features. In the right column the intra-class correlations of the respective features are shown; values lower than 0.5 indicate a poor reliability.

Feature (c=clinical; d=dermoscopic)	Intra-class correlation
Colour black (c)	0.800
Colour brown (c)	0.350
Colour grey-blue (c)	0.638
Colour light-brown (c)	0.426
Colour red (c)	0.728
Colour white (c)	0.606
Asymmetric pigmented follicular openings (d)	0.547
Irregular globules (d)	0.412
Regression/scar-like depigmentation (d)	0.241
Annular-granular pattern/grey dots (d)	0.642
Tan peripheral structureless areas (d)	0.115
Concentric circles (d)	0.681
Atypical pigment network	0.500
Blue-white veil (d)	0.710
Shiny-white structures (d)	0.777
Milky-red areas (d)	0.654
Peripheral streaks (d)	0.110
Pseudonetwork (d)	0.250
Regular globules (d)	0.655

**Table 2:** Comparison of dermoscopic features in melanomas at the ear versus nevi at the ear.

Feature	Melanomas mean (n=25)	Nevi mean (n=18)	F_(1/34)_	P≤
Asymmetric pigmented follicles	0.40	0.11	10.93	0.002
Irregular globules	0.14	0.15	0.04	n.s.
Scar-like depigmentation	0.20	0.04	7.03	0.011
Annular-granular structures	0.40	0.07	16.45	0.000
Tan peripheral structureless areas	0.16	0.01	11.54	0.002
Concentric circles	0.17	0.04	4.05	n.s.
Atypical pigment network	0.20	0.11	1.41	n.s.
Blue-white veil	0.24	0.14	1.27	n.s.
Shiny-white structures	0.29	0.12	2.67	n.s.
Milky-red areas	0.07	0.08	0.53	n.s.
Peripheral streaks	0.08	0.04	0.84	n.s.
Pseudonetwork	0.20	0.24	0.26	n.s.
Regular globules	0.03	0.22	9.60	0.004

n.s. not significant
